# Inflammatory Cytokines as Early Predictors of Weaning Failure From Extracorporeal Life Support

**DOI:** 10.1111/aor.70048

**Published:** 2025-12-03

**Authors:** Kostiantyn Kozakov, Alois Philipp, Maik Foltan, Anton Heller, Dzmitry Turtsevich, Christian Schach, Peter‐Paul Ellmauer, Walter Petermichl, Bernhard Flörchinger, Christof Schmid, Simon Schopka, Zdenek Provaznik

**Affiliations:** ^1^ Department of Cardiothoracic Surgery University Medical Center Regensburg Regensburg Germany; ^2^ Department of Internal Medicine II University Medical Center Regensburg Regensburg Germany; ^3^ Department of Anaesthesiology University Medical Center Regensburg Regensburg Germany

**Keywords:** cytokines, ECLS, interleukin‐6, interleukin‐8, V‐A ECMO, weaning failure

## Abstract

**Background:**

Weaning from extracorporeal life support (ECLS) in patients with refractory shock still remains a complex decision. Despite considerable advances in ECLS management, reliable biomarkers to predict weaning success are still not available. Inflammatory cytokines including interleukin‐6 (IL6), interleukin‐8 (IL8), and tumor necrosis factor‐alpha (TNF‐α) may reflect systemic immune response and have been proposed as potential predictors of deterioration or recovery.

**Methods:**

A retrospective, single‐center study analyzed 809 patients with ECLS between 2012 and 2024. Serum levels of IL6, IL8, and TNF‐α were measured before ECLS and 24 h after initiation. Receiver operating characteristic (ROC) analysis and subgroup comparisons between clinical phenotypes were used to assess the cytokine predictive value.

**Results:**

Weaning was achieved in 66.9% of patients. IL8 levels after 24 h demonstrated the highest predictive accuracy for weaning failure (area under the curve AUC = 0.73), outperforming IL6 and TNF‐α. The decline of IL8 levels during the first 24 h was associated (*p* = 0.008) with successful weaning. Subgroup analysis revealed that the predictive values of IL6 and IL8 were pronounced in patients with pulmonary embolism (AUC = 0.72, IL6) and septic shock (AUC = 0.77, IL8), with significantly elevated cytokine levels. Patients with structural heart disease (AUC = 0.85, IL6) and ventricular arrhythmias (AUC = 0.82, IL6) showed cytokine levels comparable to the whole cohort and a better prediction.

**Conclusion:**

Among the evaluated cytokines, IL8 exhibited the strongest predictive benefit for weaning failure, especially on Day 1. Due to its early clearance dynamics, it may be a useful parameter in the appropriate clinical situation to achieve a better outcome.

## Graphical abstract

Levels of IL‐8 and IL‐6 and their dynamics are reliable early predictors of ECLS weaning failure.

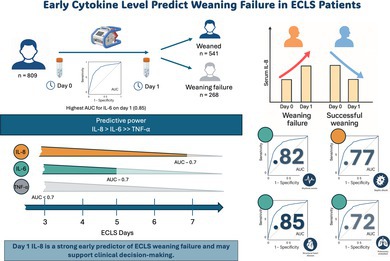

## 1 Introduction

Extracorporeal life support (ECLS) including extracorporeal membrane oxygenation (ECMO) offers a temporary lifeline for patients facing severe respiratory or cardiac failure. Although the efficacy of this approach in patients with cardiogenic shock (CS) due to acute myocardial infarction (AMI‐CS) is still under debate [1, 2, 3], it is increasingly used in patients with postcardiotomy CS (PC‐CS) [4], shock due to secondary scenarios (SC‐S) and heart failure (HF‐CS) [5]. The increase in ECLS application is attributable to advancements in technology and a better safety profile, which also led to a rise in the number of specialized centers. Despite its life‐saving potential, ECLS is still associated with relevant risks and costs, necessitating careful patient selection, vigilant monitoring, and timely weaning strategies [6, 7]. Thus, weaning from ECLS often remains a difficult clinical procedure, often guided by subjective assessments of patient improvement and various hemodynamic parameters [8].

The inflammatory response before and during ECLS is also a complex phenomenon that shares similarities with the response to cardiopulmonary bypass, but is less studied [9]. Cytokines, as key mediators of the inflammatory cascade, orchestrate a complex interplay of pro‐inflammatory and anti‐inflammatory responses that are fundamental to the pathophysiology of critical illnesses demanding ECLS. The investigation of early cytokine levels as predictive biomarkers for successful ECLS weaning represents a promising avenue for enhancing clinical decision‐making and improving patient outcomes [9, 10]. IL6 and IL8 have demonstrated predictive value in the development of organ failure [11] and outcome [10], and are available for routine clinical practice. An improved understanding of the relationship between initial cytokine profiles and ECLS weaning outcomes could enable clinicians to more accurately identify patients likely to be weaned successfully, minimize the duration of ECLS support, and reduce the incidence of associated complications.

## 2 Material and Methods

### 2.1 Study Design

An observational, single‐center, retrospective study was initiated to analyze 809 patients between 2012 and 2024, in whom TNF‐alpha, IL‐6 and IL‐8 were systematically documented before ECLS initiation and 24 h after ECLS start. We analyzed the association of cytokine levels with the time to weaning. Weaning success was defined as successful decannulation from V‐A ECMO with survival over the subsequent 24 h; all other cases were considered weaning failure. No weaning was attempted in the first 48 h after V‐A ECMO was initiated.

### 2.2 Definition of Shock Types, Clinical Scenarios

We also analyzed the predictive impact of these cytokine levels on weaning failure overall and between the patient groups: AMI‐CS—acute myocardial infarction complicated by cardiogenic shock (defined as stage C, D, or E of the Society for Cardiovascular Angiography and Interventions [SCAI] criteria) [12] including patients before, during or after revascularization (CABG or/and PCI), HF‐CS—defined as cardiogenic shock from acute decompensation of pre‐existing heart failure, without an acute myocardial infarction as the primary event [13] also including patients with DCM (dilated cardiomyopathy) as a subgroup and SC‐S—shock due to secondary scenarios: Shock due to pulmonary embolism (PE) was defined as obstructive shock with systolic blood pressure < 90 mmHg, evidence of end‐organ hypoperfusion, and hemodynamic deterioration despite vasopressor or inotropic support, requiring ECLS. Refractory arrhythmic shock (VT/VF) was defined as sustained circulatory failure due to malignant ventricular tachyarrhythmias not attributable to acute myocardial infarction, with persistent hemodynamic instability despite pharmacological management. Structural heart disease–related cardiogenic (SHD) shock was defined as acute circulatory failure due to severe valvular pathology or disease of the proximal aorta, including patients requiring ECLS before or after surgical or interventional repair. Septic shock was defined according to Sepsis‐3 [14] criteria (before 2016—Sepsis‐2) as sepsis with progressive hemodynamic deterioration under vasopressor support, requiring ECLS. Patients were considered candidates for ECLS initiation if they underwent cardiopulmonary resuscitation according to the European Resuscitation Council guidelines for at least 10 min without return of spontaneous circulation or adequate heart rhythm and were classified as an eCPR clinical scenario. In the OHCA‐eCPR scenario, ECLS was initiated in the pre‐hospital setting. Detailed information on indications and management of eCPR patients has been reported in our previous study [15]. Postcardiotomy cardiogenic shock (PC‐CS) was defined as refractory circulatory failure after cardiac surgery with inability to separate from cardiopulmonary bypass, characterized by low cardiac output (cardiac index < 2 L/min/m^2^), hypotension (systolic BP < 90 mmHg with high vasopressor dependency), and evidence of end‐organ hypoperfusion, typically requiring mechanical circulatory support.

### 2.3 Definition of Organ Failure

As all patients had undergone ECLS implantation in the sense of veno‐arterial extracorporeal membrane oxygenation (ECMO), all patients suffered from at least cardiocirculatory failure. The diagnosis of ARDS was based on the Murray Score, which includes chest x‐ray score, hypoxemia score, positive end‐expiratory pressure, expiratory system compliance. A final score of more than 2.5 established the diagnosis of ARDS [16]. Acute renal failure was defined by the parameters of the Kidney Disease Improving Global Outcomes (KDIGO) Clinical Practice Guidelines for AKI, namely Stage 3 of KDIGO criteria: serum creatinine increase > 3 times baseline, serum creatinine increases to > 4.0 mg/dL (353 μmol/L), initiation of renal replacement therapy [17]. The simultaneous occurrence of an increase of spontaneous INR more than 1.5, a threefold increase in transaminases and bilirubin more than 2.5 mg/dL defined acute hepatic dysfunction [18].

### 2.4 Management of ECLS

To establish extracorporeal life support, arterial cannulation was performed by inserting cannulas into the ascending aorta, femoral, or subclavian artery. Venous return was accomplished by placing cannulas in the femoral vein or right atrium. These cannulas were then connected to various systems, including Cardiohelp, Rotaflow (Getinge, Rastatt, Germany) and Deltastream (Xenos AG, Heilbronn, Germany), Sorin (Mirandola, Modena, Italy) pumps, as well as QUADROX Pls (Getinge, Rastatt, Germany) and HILITE (Xenos AG, Heilbronn, Germany) oxygenators.

Anticoagulation was managed with unfractionated heparin, with the goal of achieving target aPTT values of 60 s in the absence of bleeding. Flow parameters, catecholamines, and fluids were adjusted to maintain sufficient perfusion, as verified by monitoring mean arterial pressure, partial arterial oxygen pressure in the right radial artery, venous blood oxygen saturation before entering the membrane oxygenator, and serum lactate levels.

### 2.5 Analysis of Cytokines

Blood samples were collected before and 24 h after the initiation of ECLS and were promptly transported to the laboratory. IL6 levels were analyzed using electrochemiluminescence (Cobas e411, Roche Diagnostics, Rotkreuz, Switzerland), while IL8 and TNF‐alpha levels were measured by chemiluminescence (Immulite 1000, Siemens Healthcare Diagnostics, Erlangen, Germany).

### 2.6 Statistical Methods

The collected data were analyzed using IBM SPSS 29 (SPSS Inc., Chicago, IL, USA), SigmaPlot 14.0 (Systat Software Gmbh, Erkrath, Germany) and R version 4.5.0 (R Core Team, 2024). A *p*‐value of ≤ 0.05 was considered statistically significant in all analyses.

To analyze differences between two groups of patients, the Mann–Whitney *U* test for nonparametric data was chosen and a chi‐square test for categorical variables was performed. To determine differences between three or more groups, we performed an ANOVA on Ranks test. The discriminative ability of TNF‐α, IL‐6, and IL‐8 for predicting ECLS weaning failure was assessed using receiver operating characteristic (ROC) curve analysis, stratified by clinical phenotype or scenario and sampling timepoint with interpreted a priori as acceptable for values ≥ 0.70 and strong for values ≥ 0.80. Optimal cut‐offs were identified using the Youden Index (*J* = sensitivity + specificity −1). To assess the time‐dependent predictive performance of IL‐6, IL‐8 and TNF‐alpha levels measured statically on the first day during ECLS with respect to weaning failure, we performed a time‐dependent ROC curve analysis using the timeROC package in R (version 4.5.0).

## 3 Results

Among the 809 patients who received ECLS in our tertiary care center from 2012 until 2024, successful weaning was achieved in 541 individuals (66.9%), whereas 268 patients (33.1%) could not be weaned. The overall median age was 60.4 years [51.7–68.4], with no statistically significant difference between weaned and non‐weaned patients (59.7 vs. 61.2 years; *p* = 0.941). Female gender was similar between the groups (25.1% vs. 23.1%, *p* = 0.533). Body mass index (BMI) was significantly higher among patients who could not be weaned (27.5 kg/m^2^, [24.4–31.1]) compared to those who were successfully weaned (26.3 kg/m^2^, [24.1–29.3], *p* = 0.002).

The most frequent underlying diagnosis prompting ECLS initiation was acute myocardial infarction‐related cardiogenic shock (AMI‐CS), accounting for 43.8% of cases, followed by shocks due to secondary scenarios (SC‐S) causes (46.2%), including pulmonary embolism (PE), ventricular arrhythmias, septic shock, myocarditis, and structural heart disease. The distribution of major shock phenotypes, including AMI‐CS (*p* = 0.967), HF‐CS (*p* = 0.836), and SC‐S (*p* = 0.869), was balanced between groups. However, PE was significantly more common among non‐weaned patients (13.4% vs. 7.4%, *p* = 0.006). Other etiologies such as dilated cardiomyopathy, structural heart disease, myocarditis, septic shock, and post‐transplant scenarios were not significantly associated with weaning outcomes (Table 1).

**TABLE 1:** Baseline characteristics and outcome data.

Category	All patients	Weaned	Non‐weaned	*p*
Number of patients	809	541	268	
Age, years	60,4 [51,65; 68,35]	59,7 [52,15; 68,45]	61,150 [51,425; 68,075]	0.941
Women *n*, (%)	198 (24,5%)	136 (25,1%)	62 (23,1%)	0.533
BMI kg/cm^2^	26,7 [24,2; 29,5]	26,300 [24,1; 29,3]	27,5 [24,35; 31,1]	**0.002**
eCPR, patients	523 (64,6%)	332 (61,4%)	191 (71,3%)	**0.006**
OHCA‐eCPR, patients	168 (20,7%)	110 (20,3%)	58 (21,6%)	0.666
postcardiotomy, patients	177 (21,9%)	112 (20,7%)	65 (20,7%)	0.25
Follow‐up, days	48 [5; 800]	371 [42,5; 1271,5]	3 [2; 7]	**< 0.001**
Duration of ECLS, days	4 [3; 7]	5 [3; 7,5]	3 [2; 7]	**< 0.001**
In‐hospital mortality	408 (50,4%)	140 (25,9%)	268 (100%)	**< 0.001**
Neurological outcome of 555 patients				
CPC 1‐2	389 (70,1%)	389 (78,7%)	0	**< 0.001**
CPC 3‐4	11 (2,0%)	11 (2,2%)	0	**< 0.001**
CPC 5	155 (27,9%)	50 (10,1%)	105 (100%)	**< 0.001**
Multi‐organ failure	137 (16,9%)	60 (11,1%)	77 (28,7%)	**< 0.001**
AMI‐CS	**354 (43,8%)**	**237 (43,8%)**	**117 (43,6%)**	0.967
HF‐CS	**81 (10,0%)**	**55 (10,2%)**	**26 (9,7%)**	0.836
DCM	30 (3,7%)	18 (3,3%)	12 (4,5%)	0.415
SC‐S	**374 (46,2%)**	**249 (46,0%)**	**125 (46,6%)**	0.869
VT/VF	77 (9,5%)	57 (10,5%)	20 (7,5%)	0.161
PE	76 (9,4%)	40 (7,4%)	36 (13,4%)	**0.006**
Structural heart disease	74 (9,2%)	51 (9,4%)	23 (8,6%)	0.695
Septic shock	42 (5.2%)	31 (5,7%)	11 (4,1%)	0.327
Myocarditis	11 (1,4%)	9 (1,7%)	2 (0,7%)	0.289
HTx	12 (1,5%)	9 (1,7%)	3 (1,1%)	0.547
Other	82 (10,1%)	52 (9,6%)	30 (11,2%)	0.484

*Note:* Data are expressed as median and interquartile range [25th–75th] or number (percentage). Statistically significant results with *p* ≤ 0.05 are shown in bold.

Abbreviations: AKF, acute kidney failure; ALD, acute liver dysfunction; AMI‐CS, acute myocardial infarction‐related cardiogenic shock; ARDS, acute respiratory distress syndrome; BMI, Body‐Mass‐Index; CAD, coronary artery disease; CPC, cerebral performance category; DCM, dilatation cardiomyopathy; ECLS, extracorporeal life support; eCPR, extracorporeal cardiopulmonary resuscitation; eCPR‐OHCA, extracorporeal CPR following out‐of‐hospital cardiac arrest; HF‐CS, heart failure‐related cardiogenic shock; PC‐CS, postcardiotomy cardiogenic shock; PE, pulmonary embolism; SC‐S, secondary shock; VT/VF, ventricular tachycardia/ventricular fibrillation.

The proportion of patients who underwent extracorporeal cardiopulmonary resuscitation (eCPR) was notably higher in the non‐weaned group (71.3% vs. 61.4%, *p* = 0.006), whereas the occurrence of out‐of‐hospital cardiac arrest (OHCA)‐eCPR did not significantly differ between groups (*p* = 0.666). The prevalence of postcardiotomy patients was also comparable (20.7% in both groups, *p* = 0.250).

Median duration of ECLS was significantly shorter in non‐weaned patients (3 days, [2, 3, 4, 5, 6, 7]) compared to weaned patients (5 days, [3–7.5], *p* < 0.001). Accordingly, the follow‐up period in the non‐weaned cohort was likewise significantly limited (median 3 days, [2, 3, 4, 5, 6, 7]) in contrast to the weaned group (median 371 days, [42.5–1271.5], *p* < 0.001). Multi‐organ failure (MOF) occurred in 16.9% of the overall cohort during ECLS and was significantly more prevalent in the non‐weaned group (11.1% vs. 28.7%, *p* < 0.001, Table 1).

The neurological status was assessed in a subgroup of 555 patients eligible for neurological assessment. A favorable neurological outcome (CPC 1–2) was observed in 70.1%, that is, in 78.7% of weaned patients. CPC 3–4 occurred in 2.0% of patients. Cerebral death (CPC 5) was reported in 27.9% overall.

Among patients who failed to be weaned from ECLS, the leading causes were brain death (*n* = 111, 41.4%), multiorgan failure (*n* = 64, 23.9%), refractory cardiac failure (*n* = 49, 18.3%), septic circulatory collapse (*n* = 9, 3.4%), uncontrollable hemorrhage (*n* = 7, 2.6%), mesenteric ischemia (*n* = 5, 1.8%), and miscellaneous causes (*n* = 23, 8.6%).

### 3.1 Hemodynamic Profile

Hemodynamic parameters differed substantially between weaning groups. Prior to ECLS initiation, patients who were successfully weaned exhibited higher mean arterial pressure (MAP) (52 [40–62] vs. 45 [35–60] mmHg, *p* < 0.001) and lower serum lactate levels (69.5 [39–104] vs. 98.5 [60.8–140.3] mg/dL, *p* < 0.001). But any significant difference in baseline vasoactive‐inotropic score (VIS) was not observed (32.3 [12.5–59.5] vs. 30.2 [9.6–64.4], *p* = 0.807). On the first day of ECLS, MAP was comparable between groups, while lactate and VIS remained significantly lower in the weaned cohort (lactate: 24 [15–47] vs. 53 [27–92] mg/dL; VIS: 14.4 [2.1–36.7] vs. 33.0 [10.0–60.3], both *p* < 0.001, Table 2).

**TABLE 2:** Hemodynamic parameters before and during ECLS.

Category		Weaned	Not weaned	*p*
Number of patients	809	541	268	
MAP before ECLS, mmHg	50 [40; 61]	52 [40; 62]	45 [35; 60]	**< 0,001**
MAP at 1st day on ECLS mmHg	65 [60; 70]	65 [60; 70]	65 [59; 71]	0,486
Lactate before ECLS, mg/dL	78 [44,25; 119]	69,5 [39; 104]	98,5 [60,75; 140,25]	**< 0,001**
Lactate at 1st day on ECLS. mg/dL	30 [17; 62]	24 [15; 47]	53 [27; 92]	**< 0,001**
Vasoactive‐inotropic score before ECLS	31,746 [11,418; 61,279]	32,338 [12,5; 59,524]	30,19 [9,558; 64,405]	0,807
Vasoactive‐inotropic score at 1st day on ECLS	20 [3,922; 43,988]	14,386 [2,083; 36,667]	33,003 [10,0; 60,278]	**< 0,001**

*Note:* Data are expressed as median and interquartile range [25th–75th]. Statistically significant results with *p* ≤ 0.05 are shown in bold.

Abbreviations: ECLS, extracorporeal life support; MAP, mean arterial pressure.

### 3.2 Inflammatory Cytokine Levels

Non‐weaned patients exhibited higher levels of proinflammatory cytokines, both before and during ECLS. Pre‐implantation TNF‐α was modestly elevated (14 [10–24] vs. 13 [9–20] pg/mL, *p* = 0.003), which was most pronounced on the first day of support (20 [13–31] vs. 15 [11–23] pg/mL, *p* < 0.001). IL6 levels were significantly higher in non‐weaned patients at both time points—480 [146–1775.5] vs. 274 [4–780] pg/mL pre‐ECLS and 549.5 [185–2317.8] vs. 155 [71–444.5] pg/mL on day one (both *p* < 0.001). Similarly, IL8 was elevated in the non‐weaned group before (144 [53–390.3] vs. 83 [37–233] pg/mL) and during ECLS (184 [79–734] vs. 64 [36.8–137] pg/mL; both *p* < 0.001). Patients successfully weaned from ECMO exhibited a decline in IL6 and IL8 levels within 24 h, indicative of effective cytokine clearance and a resolving inflammatory response. Conversely, patients experiencing weaning failure showed either rising (IL8 and TNF‐alpha) or stable (IL6) cytokine levels, suggesting impaired clearance and ongoing systemic inflammation (Table 3).

**TABLE 3:** Analysis of interleukins between patients.

	Weaned	Not weaned	*p*
Preimplantation TNF‐alpha, pg/mL	13 [9; 20]	14 [10; 24]	**0,003**
1st day TNF‐alpha, pg/mL	15 [11; 23]	20 [13; 31]	**< 0,001**
*p‐value before* versus *24 h on ECLS*	**< 0,001**	**< 0,001**	
Preimplantation IL6, pg/mL	274 [4; 780]	480 [146; 1775,5]	**< 0,001**
1st day IL6, pg/mL	155 [71; 444,5]	549,5 [185; 2317,75]	**< 0,001**
*p‐value before* versus *24 h on ECLS*	**< 0,001**	0.249	
Preimplantation IL8, pg/mL	83 [37; 233]	144 [53; 390,25]	**< 0,001**
1st day IL8, pg/mL	64 [36,75; 137]	184 [79; 734]	**< 0,001**
*p‐value before* versus *24 h on ECLS*	**0,008**	**0,008**	

*Note:* Data are expressed as median and interquartile range [25th–75th]. Statistically significant results with *p* ≤ 0.05 are shown in bold.

Abbreviations: IL6, interleukin‐6; IL8, interleukin‐8; TNF‐alpha, tumor necrosis factor alpha.

### 3.3 Predictive Performance of Cytokines for ECLS Weaning Failure Across Phenotypes

Among the most prevalent clinical scenarios, both AMI‐CS and HF‐CS exhibited modest AUCs for IL6 and IL8 at baseline (approximately 0.60), with consistent improvement in 24 h. IL8 reached AUCs of 0.737 in AMI‐CS and 0.714 in HF‐CS, while IL6 improved to 0.698 and 0.650, respectively.

Across most phenotypes, TNF‐α yielded lower AUC values than IL‐6 and IL‐8, with no subgroup showing high predictive accuracy. The highest AUC observed for TNF‐α was 0.695 in HF‐CS on Day 1, whereas pre‐ECLS values often remained below 0.60.

#### 3.3.1 Refractory Rhythmic Events

Overall, the predictive performance of cytokines improved from pre‐implantation to Day 1 of ECLS, particularly for IL6 and IL8 (Table 4). Patients with refractory rhythmic events: ventricular tachycardia and ventricular fibrillation (VT/VF) demonstrated high discriminative accuracy at baseline (IL‐6 AUC 0.764, IL‐8 AUC 0.770), with further increases in 24 h (0.821 and 0.814, respectively). In this subgroup of patients, ROC curve analysis identified an optimal cut‐off of 343.5 pg/mL for IL‐6, resulting in a sensitivity of 75.0%, specificity of 77.2%, and a Youden Index of 0.522. For IL‐8, the optimal threshold was 98.5 pg/mL, yielding a sensitivity of 80.0%, specificity of 69.1%, and a Youden Index of 0.491.

**TABLE 4:** Predictive performance of cytokines for ECLS weaning failure across phenotypes.

Groups	Timepoint	TNF‐α AUC	IL‐6 AUC	IL‐8 AUC
All patients	Pre‐ECLS	0.570	0.600	0.594
	Day 1	0.618	0.706	0.732
AMI‐CS	Pre‐ECLS	0.618	0.623	0.624
	Day 1	0.663	0.698	0.737
HF‐CS	Pre‐ECLS	0.535	0.535	0.604
	Day 1	0.695	0.650	0.714
DCM^a^	Pre‐ECLS	0.435	0.599	0.608
	Day 1	0.597	0.773	0.852
SC‐S	Pre‐ECLS	0.548	0.599	0.583
	Day 1	0.583	0.722	0.733
PE	Pre‐ECLS	0.448	0.410	0.407
	Day 1	0.568	0.723	0.692
VT/VF	Pre‐ECLS	0.680	0.764	0.770
	Day 1	0.691	0.821	0.814
Septic shock	Pre‐ECLS	0.592	0.611	0.690
	Day 1	0.687	0.712	0.768
Structural heart disease	Pre‐ECLS	0.639	0.754	0.634
	Day 1	0.606	0.846	0.793
Other	Pre‐ECLS	0.568	0.594	0.516
	Day 1	0.584	0.608	0.643

Abbreviations: AMI‐CS, acute myocardial infarction‐related cardiogenic shock; AUC, area under the curve; Day 1, first day after ECLS initiation; DCM, dilated cardiomyopathy; ECLS, extracorporeal life support; HF‐CS, heart failure‐related cardiogenic shock; IL‐6, interleukin‐6; IL‐8, interleukin‐8; PC‐CS, postcardiotomy cardiogenic shock; PE, pulmonary embolism; Pre‐ECLS, before initiation of ECLS; SC‐S, secondary cardiogenic shock; TNF‐α, tumor necrosis factor alpha; VT/VF, ventricular tachycardia/ventricular fibrillation.

^a^ Due to the small number of patients, the observed results may be subject to sampling bias and should be considered exploratory rather than definitive.

#### 3.3.2 Structural Heart Disease

A similar trend was observed in structural heart disease (SHD), where IL6 on Day 1 achieved an AUC of 0.846, accompanied by strong performance from IL8 (AUC–0.793). In the subgroup of patients with structural heart disease the optimal cut‐off for IL‐6 was 210.0 pg/mL, which yielded a sensitivity of 95.7%, specificity of 64.7%, and a Youden Index of 0.604. IL8 levels showed a comparable predictive profile, with an optimal cut‐off of 93.5 pg/mL corresponding to a sensitivity of 76.2%, specificity of 73.5%, and a Youden Index of 0.497.

#### 3.3.3 Septic Shock

In the septic shock subgroup, IL8 showed a notable increase in predictive power from 0.690 pre‐ECLS to 0.768 in 24 h, with parallel improvements in IL6 (0.611–0.712). The optimal cut‐off value of IL8 on the first day was 161.5 pg/mL, corresponding to a sensitivity of 90.9%, a specificity of 58.1%, and a Youden Index of 0.490.

#### 3.3.4 Pulmonary Embolism

Patients with pulmonary embolism (PE) had poor cytokine‐based prediction at baseline (IL‐6 and IL‐8 AUCs~0.41), though performance improved by Day 1 (0.723 and 0.692, respectively).

In addition to primary diagnostic categories, analysis of ECLS subgroups revealed further differences in biomarker performance between different clinical scenarios (Table 5).

**TABLE 5:** Predictive performance of cytokines for ECLS weaning failure related to clinical scenario.

Groups	Timepoint	TNF‐α AUC	IL‐6 AUC	IL‐8 AUC
eCRP	Pre‐ECLS	0.601	0.606	0.595
	Day 1	0.657	0.710	0.748
eCPR‐OHCA	Pre‐ECLS	0.713	0.691	0.652
	Day 1	0.724	0.783	0.780
PC‐CS	Pre‐ECLS	0.605	0.637	0.626
	Day 1	0.590	0.716	0.745

Abbreviations: AUC, area under the curve; eCPR, extracorporeal cardiopulmonary resuscitation; eCPR‐OHCA, extracorporeal CPR following out‐of‐hospital cardiac arrest; IL‐6, interleukin‐6; IL‐8, interleukin‐8; PC‐CS, postcardiotomy cardiogenic shock; TNF‐α, tumor necrosis factor alpha.

#### 3.3.5 Extracorporeal Cardiopulmonary Resuscitation

In the overall eCPR cohort, the discriminatory performance of IL‐6 and IL‐8 improved from baseline (AUC, 0.606 and 0.595, respectively) to 24 h (AUC, 0.710 and 0.748). For TNF‐α, the AUC increased from 0.601 at baseline to 0.657 at 24 h. Patients undergoing eCPR following OHCA exhibited the strongest predictive profiles on Day 1, with IL‐6 and IL‐8 achieving AUCs of 0.783 and 0.780, respectively. TNF‐α was stable across timepoints (0.713 and 0.724). In this subgroup of patients (eCPR‐OHCA), both IL‐6 and IL‐8 levels on the first day of ECLS demonstrated strong predictive accuracy for weaning failure. The optimal threshold for IL‐6 was 268.5 pg/mL, resulting in a sensitivity of 77.2%, specificity of 70.4%, and a Youden Index of 0.476. For IL‐8, the best‐performing cut‐off was 100.5 pg/mL, which yielded a sensitivity of 77.8%, specificity of 64.8%, and a Youden Index of 0.426.

#### 3.3.6 Postcardiotomy Cardiogenic Shock

Among patients with postcardiotomy cardiogenic shock (PC‐CS), IL6 and IL8 showed improved performance on Day 1 (0.716 and 0.745), compared to 0.637 and 0.626 pre‐ECLS. In contrast, TNF‐α demonstrated limited discriminative ability in this subgroup, with no improvement over time (0.605 vs. 0.590). The optimal cut‐off of IL8 on the first day was 52.5 pg/mL, yielding a sensitivity of 88.5%, specificity of 47.2%, and a Youden Index of 0.357.

**FIGURE 1:**
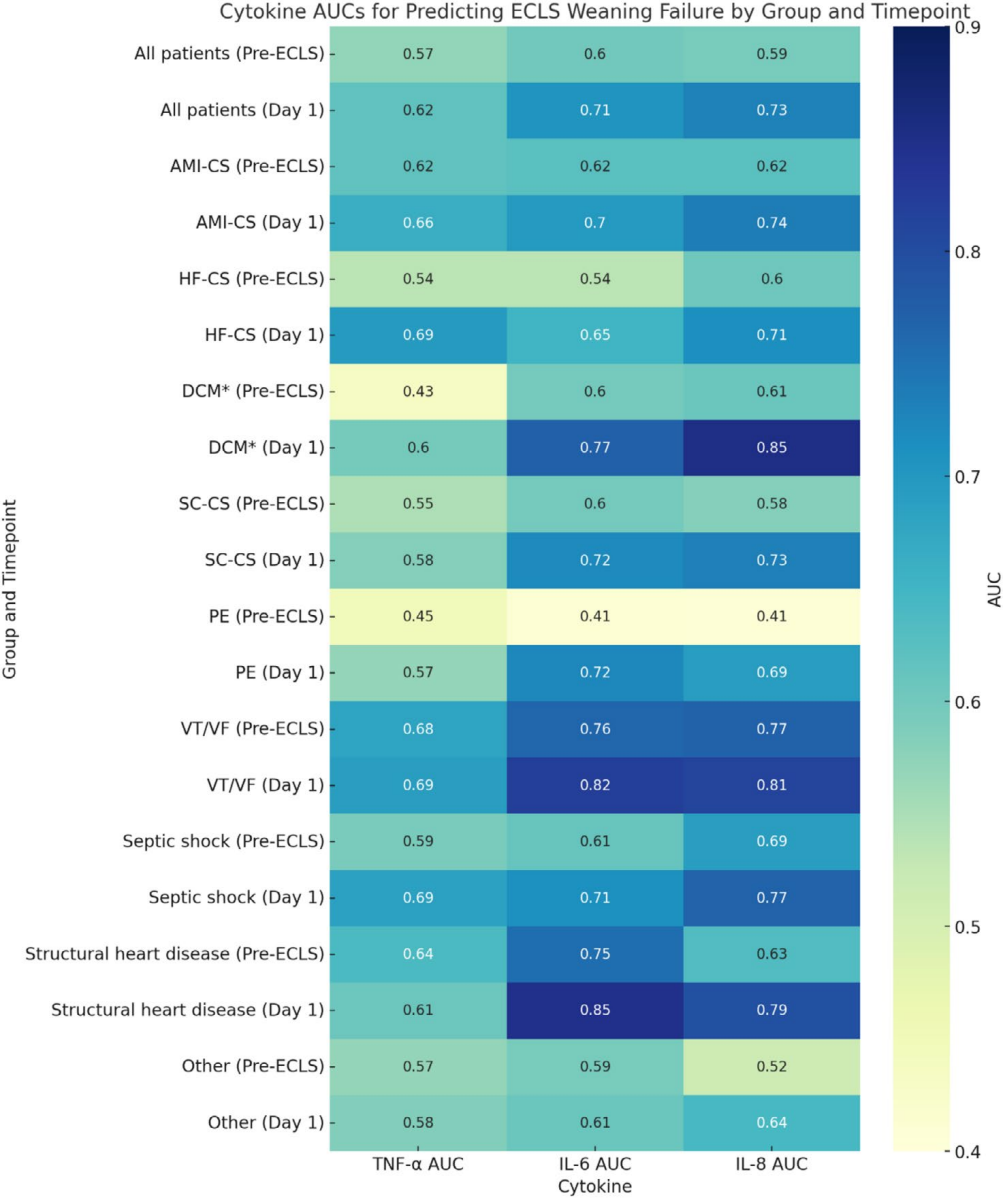
Double‐Row Heatmap of cytokine AUSs for predicting ECLS weaning failure. AMI‐CS, acute myocardial infarction‐related cardiogenic shock; AUC, area under the curve; Day 1, first day after ECLS initiation; DCM, dilated cardiomyopathy; ECLS, extracorporeal life support; eCPR, extracorporeal cardiopulmonary resuscitation; eCPR‐OHCA, extracorporeal CPR following out‐of‐hospital cardiac arrest; HF‐CS, heart failure‐related cardiogenic shock; IL‐6, interleukin‐6; IL‐8, interleukin‐8; PC‐CS, postcardiotomy cardiogenic shock; PE, pulmonary embolism; Pre‐ECLS, before initiation of ECLS; SC‐CS, secondary cardiogenic shock; TNF‐α, tumor necrosis factor alpha; VT/VF, ventricular tachycardia/ventricular fibrillation. * Due to the small number of patients, the observed results may be subject to sampling bias and should be considered exploratory rather than definitive. [Color figure can be viewed at wileyonlinelibrary.com]

A detailed summary of AUC values by phenotype and timepoint is presented in Tables 4 and 5, Figure 1.

#### 3.3.7 Comparison of Cytokine Levels Between Clinical Subgroups: SHD, Septic Shock, PE, VT/VF

To determine the probable reason for the different predictive power among the groups, we performed an ANOVA on Ranks test (Table 7). ECLS duration did not differ significantly between AMI, SC, and HF groups (*p* = 0.183). But the values of IL6 and IL8 differed significantly among groups (Kruskal–Wallis *p* < 0.001 for both). SC‐S patients had the highest median levels for IL6 (264.5 pg/mL) and IL8 (108.0 pg/mL), followed by AMI (202.5 and 74.0 pg/mL, respectively) and HF (104.0 and 64.0 pg/mL, respectively). Post hoc analysis confirmed significantly higher cytokine levels in SC versus HF and SC versus AMI, while differences between AMI and HF were not significant.

Therefore, we decided to analyze the SC subgroup and compare the levels of cytokines with high predictive AUC value for weaning failure and the rest of the cohort. Cytokine profiles on the first day of ECLS differed across clinical subgroups. Patients with septic shock demonstrated significantly elevated IL6 (1378 [208.3; 65462.5] pg/mL) and IL8 levels (206 [76.8; 7945.3] pg/mL) compared to others (*p* < 0.001 for both). Similarly, patients with pulmonary embolism (PE) exhibited higher IL6 (270 [125.5; 1393.3] pg/mL, *p* = 0.044) and IL8 levels (124 [55.0; 849.0] pg/mL, *p* = 0.013) than those without. In contrast, no statistically significant differences were observed in IL6 or IL8 levels between patients with structural heart disease (SHD) and those without (*p* = 0.964 and *p* = 0.602, respectively). Likewise, patients with refractory rhythmic events showed comparable cytokine levels to the rest of the cohort (IL‐6: 174 [68.5; 886.0] vs. 233 [91.0; 744.3] pg/mL, *p* = 0.209; IL‐8: 83 [34.0; 258.0] vs. 86 [44.0; 242.5] pg/mL, *p* = 0.278).

### 3.4 Weaning Time

**FIGURE 2:**
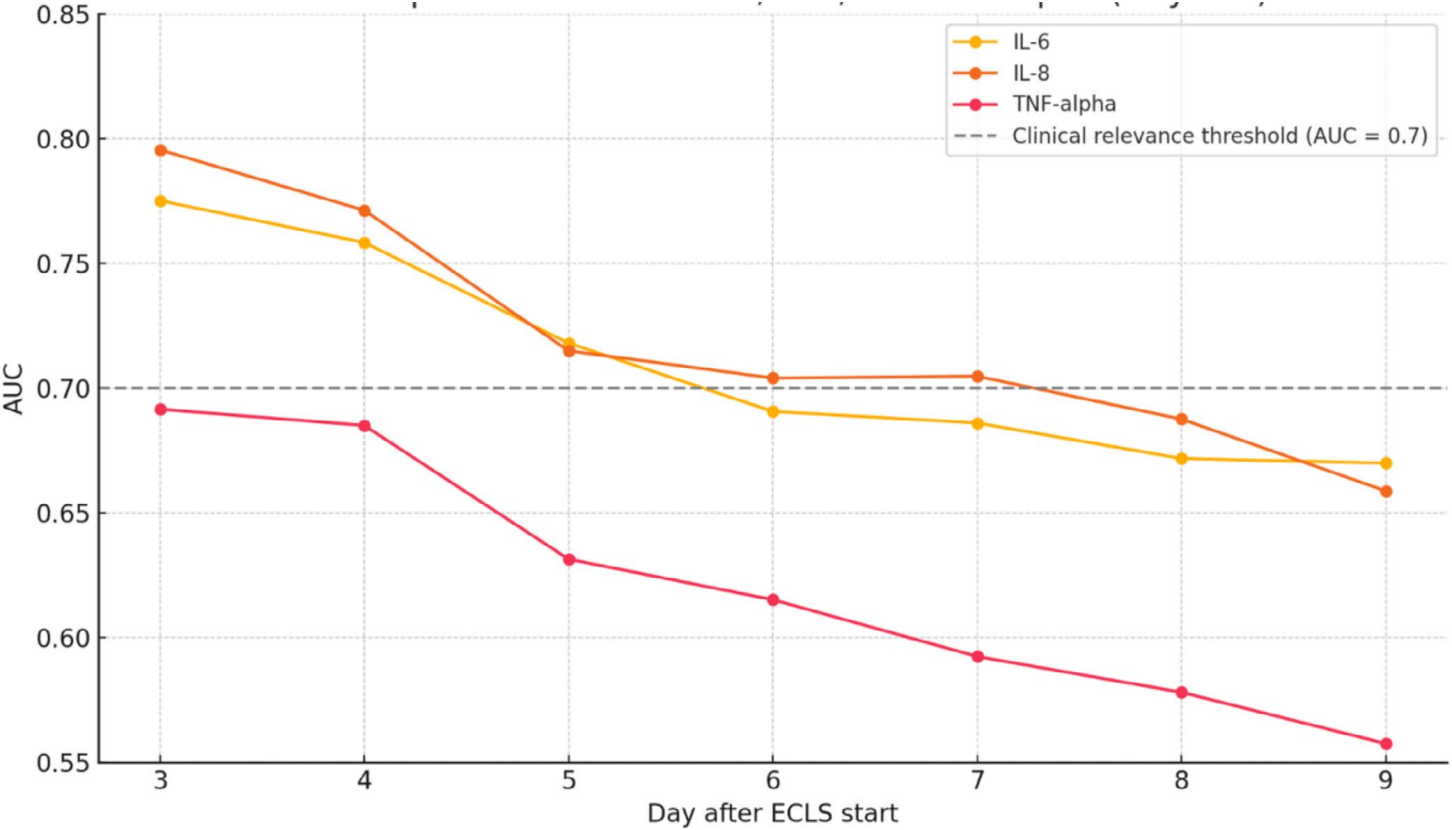
Prognostic performance of day‐one cytokines for ECLS weaning failure across the time during support. AUC, area under the curve; IL‐6, interleukin‐6; IL‐8, interleukin‐8; TNF‐α, tumor necrosis factor alpha. [Color figure can be viewed at wileyonlinelibrary.com]

Since the data demonstrated different levels of predictive power in the patient groups, we were interested in assessing the predictive power of cytokine levels on the time of ECLS weaning. Time‐dependent ROC analysis was used to assess the predictive accuracy of IL‐6, IL‐8, and TNF‐α levels measured on Day 1 after ECLS initiation for weaning failure across Days 3–9. IL‐8 demonstrated the highest overall predictive performance, with an AUC of 0.796 on Day 3 and remaining above the clinical relevance threshold of 0.70 until Day 7. IL‐6 also showed acceptable discriminatory ability, reaching an AUC of 0.775 on Day 3 and maintaining AUCs > 0.70 until Day 5. In contrast, TNF‐α exhibited lower predictive accuracy throughout, with a maximum AUC of 0.692 on Day 3 and a progressive decline below 0.60 by Day 7 (Table 6; Figure 2).

**TABLE 6:** Time‐dependent AUC values for IL‐6, IL‐8, and TNF‐α measured on Day 1 after ECLS initiation in predicting weaning failure across Days 3 and 9.

Day	IL‐6 AUC	IL‐8 AUC	TNF‐alpha AUC
3	0.775	0.795	0.692
4	0.758	0.771	0.685
5	0.718	0.715	0.631
6	0.691	0.704	0.615
7	0.686	0.705	0.592
8	0.672	0.688	0.578
9	0.67	0.659	0.558

Abbreviations: AUC, area under the curve; IL‐6, interleukin‐6; IL‐8, interleukin‐8; TNF‐α, tumor necrosis factor alpha.

**TABLE 7:** Comparison of IL‐6 and IL‐8 levels on the first day of ECMO support between specific clinical subgroups (SHD, septic shock, PE, VT/VF) and the rest of the cohort.

Subgroup	Cytokine	Median [IQR]	Rest median [IQR]	*p*
SHD	IL‐6	242.5 [96.75; 516.25]	229 [87; 789]	0.964
SHD	IL‐8	74.5 [46; 183.75]	87 [43; 258]	0.602
Septic shock	IL‐6	1378.0 [208.25; 65462.5]	218 [84; 690]	**< 0.001**
Septic shock	IL‐8	206.0 [76.75; 7945.25]	82 [43; 227]	**< 0.001**
PE	IL‐6	270.0 [125.5; 1393.25]	228 [84.5; 725.5]	**0.044**
PE	IL‐8	124.0 [55; 849]	83 [43; 223.5]	**0.013**
VT/VF	IL‐6	174.0 [68.5; 886]	233 [91; 744.25]	0.209
VT/VF	IL‐8	83.0 [34; 258]	86 [44; 242.5]	0.278

*Note:* Median values with interquartile ranges [25%; 75%] are shown. Statistically significant results with *p* ≤ 0.05 are shown in bold.

Abbreviations: IL‐6, interleukin‐6; IL‐8, interleukin‐8; PE, pu; SHD, structural heart disease; TNF‐α, tumor necrosis factor alpha; VT/VF, ventricular tachycardia/ventricular fibrillation.

To evaluate the predictive performance of first‐day IL6 and IL8 across all subgroups of patients, we calculated the area under the ROC curve for those who were either successfully weaned or experienced weaning failure within the first 5 days of ECLS. The AUC for IL6 on Day 1 was 0.758, with the highest Youden Index (*J* = 0.209) observed at a cut‐off value of 71.5 pg/mL, yielding a sensitivity of 0.934 and specificity of 0.275. In comparison, IL8 demonstrated superior predictive accuracy with an AUC of 0.800. The optimal cut‐off for IL8 was 99.5 pg/mL, corresponding to a Youden Index of 0.488, with sensitivity and specificity of 0.769 and 0.719, respectively. These results suggest that IL8 may provide a better overall balance between sensitivity and specificity for early prediction of weaning failure across all patient subgroups.

## 4 Discussion

The decision to wean patients from ECLS, as well as the weaning procedure itself, continues to be a challenge for clinicians. Risk scores such as the Survival After Veno‐arterial ECMO score (SAVE, ROC AUC = 0.77 (95% CI 0.69–0.86), *p* < 0.001) [19] or the Prediction of Cardiogenic Shock Outcome for AMI Patients Salvaged by V‐A ECMO score (ENCOURAGE, ROC AUC = 0.84 (95% CI 0.77–0.91)) [20] have been developed to facilitate decision‐making. Despite initial skepticism regarding the use of biomarkers to assess weaning from ECLS [21], several studies demonstrated a valuable predictive power of biomarkers: lactate clearance with OR = 0.5 (95% CI 0.3–0.9, *p* = 0.02), serum lactate at 24 h with ROC AUC = 0.84 in AMI‐CS for successful weaning [22, 23, 24]. In our center, we could recently demonstrate the predictive power of IL6 and IL8 on patient outcome [10]. In relation to weaning from other life‐sustaining therapies, elevated circulating IL‐6 and IL‐8 in mechanically ventilated patients are associated with a greater likelihood of weaning failure and a substantially increased risk of reintubation [25, 26]. Beyond the ventilatory domain, higher levels of these cytokines are likewise linked to greater vasopressor requirements and worse early outcomes among patients with post–cardiac‐surgery vasoplegic syndrome [27, 28]. In the present study, we investigated the impact of cytokines as predictors for ECLS weaning and their pathophysiological role during V‐A ECMO in more detail. The predictive power of IL6, IL8 and TNF‐alpha was evaluated for weaning failure in different clinical scenarios and shock phenotypes. To our best knowledge, there were no studies of this size to evaluate ECLS weaning from the perspective of cytokines in the dynamics and across different clinical entities.

Comparison of clinical parameters associated with weaning failure in ECLS patients demonstrated, as in previous investigations, a potential association between higher BMI and reduced chances of successful weaning [29, 30], as well as the role of previous cardiac arrest as potent determinants of ECLS weaning failure [31]. Patient age did not significantly impact weaning success. While prior studies link advanced age to poorer outcomes [31], recent analyses favor biological age or frailty as better markers. Our data implies that appropriately selected older patients can achieve comparable weaning outcomes [32].

The incidence of MOF was significantly higher among non‐weaned patients, and probably even underestimated, as it requires enough time for the manifestation of MOF during ECLS. It remains one of the strongest predictors of both mortality and weaning failure in ECMO patients [11, 31]. Its presence reflects advanced disease severity and systemic inflammation, hallmarks of a mainly irreversible deterioration that requires early identification and aggressive management to improve weaning potential.

Neurological status remains a critical factor with regard to ECLS weaning too. Preserved cerebral function is often a key element for successful decannulation, highlighting the importance of timely recognition of irreversible brain injury. Early identification and prompt intervention in patients with potentially reversible neurologic impairment are essential to optimize outcomes [8, 33].

Hemodynamic parameters and metabolic changes proved to be clear predictors of ECLS patients' weaning [24]. Pre‐ECLS hypotension and significantly high levels of lactate in the weaning‐failure cohort, reflect profound shock and impaired perfusion. These findings are supported by previous studies showing that elevated lactate levels before ECLS initiation are independent predictors of mortality and unsuccessful weaning [24, 34]. Moreover, a high VIS during early ECLS support was associated with poor outcomes, consistent with previous studies where an elevated VIS demonstrated a higher mortality rate in cardiogenic shock [31]. To summarize, the degree of pre‐ECLS shock and the early response to support, especially regarding lactate clearance and vasopressor dependence, are key determinants of weaning success.

Our findings demonstrate a clear association between early inflammatory cytokine dynamics and weaning outcomes in patients with ECLS. Patients who were successfully weaned exhibited a significant reduction in IL6 and IL8 levels within the first 24 h of support, suggesting a favorable immunologic response and effective cytokine clearance. In contrast, non‐weaned patients showed stable or increasing levels of cytokines, indicating a sustained or worsening systemic inflammatory response and insufficient cytokine clearance despite mechanical circulatory support. The divergent cytokine kinetics support the hypothesis that early modulation of inflammation is essential for recovery during ECLS. These findings highlight early cytokine clearance as a potential predictor of successful ECLS weaning and underline the value of dynamic biomarker monitoring to guide clinical decision‐making.

In our study IL6 and IL8 levels demonstrated the highest predictive performance for weaning failure within 24 h after ECLS initiation. This finding is in contrast to the pre‐implantation values indicating that dynamic changes rather than baseline levels better reflect the evolving pathophysiology and clinical relevance under extracorporeal support. In this regard, it is also important to note the kinetics of cytokines. TNF‐α appears in the bloodstream rapidly, with peak levels typically being reached within 1–2 h after an inflammatory stimulus and is followed by a rapid decline due to its short half‐life of 20–30 min [35]. In contrast, IL6 and IL8 show a slightly delayed, but sustained response, with levels rising within 2–3 h and peaking at approximately 4–6 h, often remaining elevated for 24 h or longer depending on the severity of the systemic inflammation [36, 37]. Furthermore, it is important to note that after the initiation of ECLS, a number of various procedures (revascularization, interventions at the intensive care unit, diagnostic procedures) and pathophysiological processes (organ reperfusion) occur within the first hours, which are also considerably decisive in the further course and thus affect the level of inflammation and determine the predictive power of the cytokines, explicitly in 24 h on ECLS.

In light of the evolving clinical course during ECLS, we sought to investigate the prognostic utility of cytokine levels after 24 h on ECLS for weaning failure at different time points throughout the support. The temporal analysis of cytokine predictive performance revealed that IL6 and IL8 levels measured 24 h after ECLS initiation exhibit strong prognostic value for subsequent weaning failure, particularly in the early phase of support. Both cytokines demonstrated their highest discriminatory power on Day 3, with a gradual decline in predictive accuracy over the following days (Figure 2). This trend underscores the importance of an early inflammatory burden as a determinant of the clinical course and suggests that cytokine levels reflect an evolving pathophysiological process most relevant within the initial days of extracorporeal support. The progressive decline in early cytokines (e.g., Day‐1 IL‐6 and IL‐8) predictive power over time reflects the dynamic course of ECLS, wherein the early inflammatory ‘shock‐hit’ is subsequently shaped by ongoing therapeutic interventions, and, as support continues, the secondary impact of possible complications and interventions such as infection, hemorrhage, the need for renal replacement therapy, etc. emerge as dominant determinants of clinical trajectory, potentially attenuating the prognostic relevance of early cytokine levels.

Considering the decline in AUC values over the time of ECLS, we analyzed the subgroups and did not find a significant difference in the duration of ECLS between the subgroups. Furthermore, the subgroups with the best interleukin predictive power, such as patients with refractory arrhythmias or structural heart disease, did not differ in terms of cytokine level from the overall cohort. At the same time, in patients with high, but slightly lower relevant predictive value, such as pulmonary embolism or septic shock, the levels of predictive cytokines (IL6 and IL8 on the first day) were significantly higher compared to the whole cohort. Here we can suggest that these patients have a different pathophysiologic type of shock and therefore demonstrate higher values. We also found a significantly higher cut‐off of IL8 in the prediction of weaning failure by patients with septic shock, which is most likely caused by an additional initial septic trigger (infection).

Another interesting finding is the more pronounced predictive ability of OHCA eCPR patients to weaning failure compared to the overall eCPR cohort, which is most likely due to the lack of preexisting inflammatory burden in this clinical scenario, as we have already noted in previous studies [15].

Although the investigated cytokines are not absolute predictors of weaning failure, certain markers—particularly under specific clinical scenarios—demonstrate predictive accuracy comparable to established multi‐component outcome scores [20]. Among them, IL8 measured on the first day of ECLS may serve as a valuable adjunct in clinical decision‐making and has the potential to complement future prognostic scoring systems. Moreover, the cut‐off values identified in this study could establish a base for future trials evaluating immunomodulatory strategies tailored to specific pathophysiological states—such as revisiting the indications for CytoSorb therapy or defining thresholds for initiating therapeutic plasma exchange.

## 5 Limitations

Possible limitations of this study are the period of 12 years during which data were collected, considering changing features of ECLS patient management and technical tools over time. Furthermore, the underlying patient population presents rather heterogeneous. The retrospective nature of the analysis as well as reporting the experience of a single center may also be a restriction for definitive conclusions. As an observational study, our findings are hypothesis‐generating and require validation in future prospective studies. Due to multiple testing, a risk of type I errors cannot be excluded.

## 6 Conclusion

IL6 and, more notably, IL8 levels measured in 24 h after initiation of ECLS demonstrate substantial discriminatory power for the early prediction of weaning failure and should be estimated in the appropriate clinical situation to achieve better predictive utility. The prognostic utility of these biomarkers declines over the duration of ECLS and appears to be influenced by other specific clinical factors during the ECLS.

## Author Contributions

Kozakov participated in the design of the study, was the main coordinator of the study, performed the statistical analysis, and revised the manuscript. Philipp and Foltan participated in the coordination of the study, collected data and revised the manuscript. Heller, Schach, Turtsevich, Ellmauer, Petermichl and Flörchinger collected data and helped to draft the manuscript. Schmid and Schopka participated in the design and coordination of the study, helped to draft the manuscript and revised the manuscript. Provaznik supervised the study, participated in the design and coordination of the study and helped to draft the manuscript. All authors read and approved the final manuscript.

## Ethics Statement

The study was conducted according to the principles of the Declaration of Helsinki and was approved by the Ethics Committee of the University Medical Center of Regensburg (Reference number: 25‐4238‐104; 10. June 2025).

## Conflicts of Interest

The authors declare no conflicts of interest.

## Data Availability

The datasets used and/or analyzed during the current study are available from the corresponding author on reasonable request.
